# Efficacy of focused low-intensity pulsed ultrasound therapy for the management of knee osteoarthritis: a randomized, double blind, placebo-controlled trial

**DOI:** 10.1038/srep35453

**Published:** 2016-10-17

**Authors:** Lang Jia, Yan Wang, Jinyun Chen, Wenzhi Chen

**Affiliations:** 1 Department of Rehabilitation Medicine, the Second Affiliated Hospital of Chongqing Medical University, 74 Linjiang Road, Chongqing 40010, China; 2 State Key Laboratory of Ultrasound Engineering in Medicine Co-Founded by Chongqing and the Ministry of Science and Technology, College of Biomedical Engineering, Chongqing Medical University, Chongqing 400016, China; 3 Clinical Center for Tumor Therapy, the Second Affiliated Hospital of Chongqing Medical University, 74 Linjiang Road, Chongqing 400010, China

## Abstract

The aim of this study was to investigate the effects of focused low-intensity pulsed ultrasound (FLIPUS) therapy on the functional and health status of patients with knee osteoarthritis (KOA). A total of 106 subjects with bilateral KOA were randomized sequentially into two groups. Group I received FLIPUS + diclofenac sodium, and group II received sham FLIPUS + diclofenac sodium. The therapeutic effects of the interventions were evaluated by measuring changes in VAS pain, the WOMAC scores, and the LI scores after 10 days of treatment as well as changes in LI and VAS at follow-up, 4 and 12 weeks later. In addition, changes in the range of motion, ambulation speed, and the SF-36 in each group were recorded after 10 days of treatment. Compared with those in group II, patients in group Ishowed significant improvements in VAS, WOMAC, LI, ambulation speed, and most items in the SF-36 after 10 days of treatment. In addition, patients in group I showed significant improvements in LI and VAS at follow-up. There were no FLIPUS-related adverse events during and after the interventions. In conclusion, FLIPUS is a safe and effective treatment modality for relieving pain and improving the functions and quality of life of patients with KOA.

Osteoarthritis (OA), the most common of all arthritic conditions, is characterized by joint pain and stiffness. It is a common and significant chronic musculoskeletal disease that reduces mobility and has a considerable impact on quality of life. Over 50% of people over the age of 65 years have radiological evidence of the disease, and approximately 10% of males and 18% of females have symptomatic knee OA^1^. Therefore, knee OA is likely to become the fourth most important global cause of disability in females and the eighth most important cause in males^2^.

Although various management techniques are available for the treatment of OA, there are presently no therapies that modify the onset or progression of OA-induced structural damage^3^,^4^. The aims of managing knee OA are to relieve pain, delay complications, and prevent disease progression. Clinical guidelines for managing knee OA, which have been published by the American College of Rheumatology (ACR), the American Academy of Orthopedic Surgeons (AAOS), and the European League Against Rheumatism (EULAR), recommend conservative treatments (i.e., self-management programs, strengthening, low-impact aerobic exercises, weight loss, and neuromuscular education) as well as pharmacologic treatments (i.e., nonsteroidal anti-inflammatory drugs and tramadol)^5^,^6^. Surgery is reserved for patients whose symptoms have not responded to other treatments. These recommended methods are not perfect and have many disadvantages: they often are expensive and invasive if surgery is involved, and the adverse effects of NSAIDs are cause for concern. Therefore, innovative and cost-effective approaches that can prevent the development and progression of OA are urgently needed.

Ultrasound (US) treatment has been used as a non-invasive modality for the management of OA for more than 60 years because of its reputed ability to relieve pain^7^, reduce edema, increase the range of motion, and accelerate tissue repair^8^ via thermal and non-thermal mechanisms (mechanical effects). US can be administered in either a continuous or a pulsed mode. Pulsed US produces non-thermal effects and is beneficial for cartilage health^9^,^10^,^11^,^12^,^13^,^14^, whereas continuous US aims to generate thermal effects that could enhance fibrous tissue extensibility, increase tissue metabolism, promote capillary permeability, and elevate the pain threshold^15^,^16^,^17^,^18^. A recent systematic review and meta-analysis suggested that pulsed US is the preferred treatment mode both in terms of more effective pain relief and improved function without significant adverse effects in clinical trials^19^. In addition, US can be administered in either an unfocused or a focused mode. The basic differences between FLIPUS and traditional US are that the main biological effect of FLIPUS is a mechanical effect and the targeted tissue is cartilage, while the biological effect of traditional US is a thermal effect and the targeted tissues are periarticular soft tissue lesions. The results of a number of studies have suggested that unfocused therapeutic US may be useful for reducing the pain and disability associated with knee OA^20^,^21^,^22^,^23^,^24^. However, few studies of focused low-intensity pulsed US(FLIPUS) have been published that describe knee OA rehabilitation.

Therefore, the purpose of this study was to elucidate the effects of FLIPUS for the management of knee OA. The aim of this prospective randomized double-blind placebo-controlled trial was to evaluate the short-term effectiveness of FLIPUS therapy on pain, physical function, ambulation activity, and health-related quality of life (HRQoL) in patients with knee OA.

## Subjects and Methods

### Subjects

This prospective randomized placebo-controlled clinical trial was approved by the Institutional Review Board and Hospital Research Ethics Committee of Chongqing Medical University, on April 3, 2014 (approval no. 2014005), and was conducted at the Department of Rehabilitation Medicine, the Second Affiliated Hospital, Chongqing Medical University, Chongqing, China between Feb 2015 and Feb 2016. The Chinese Clinical Trial Registry (a non-profit organization, established according to both the WHO International Clinical Trials Register Platform Standard and Ottawa Group Standard)granted full approval of the study protocol, recruitment materials, and consent form (http://www.chictr.org.cn; Registration NO. ChiCTR-IPR- 14005748; Date of registration.2014-12-26). All methods were carried out in accordance with the approved ethical guidelines. After the study had been completely described to the participants, they all signed written informed consent forms.

Each participant was initially interviewed and evaluated by an attending orthopedist, and the evaluation was then confirmed by a well-trained and experienced research team physiatrician. The inclusion criteria were as follows: age ≥ 40 years, knee OA fulfilling the ACR classification criteria^25^, Kellgren & Lawrence class rating of II, and III^26^, knee pain, and limitation on most days within the past 6 months. The exclusion criteria were as follows: rheumatoid arthritis, gouty arthritis, infectious arthritis, a history of knee joint replacement on the study knee, current or past (within 6 months) oral or intra-articular corticosteroid use, physiotherapy, acupuncture treatment, the use of exercises specifically for the knee within the past 6 months, a medical condition that precludes safe exercise (such as uncontrolled hypertension, a heart condition, hematological diseases coagulopathy, gastrointestinal ulcers, or a hemorrhage), a history of taking NSAIDs or symptomatic slow-acting drugs for OA (diacerein, hyaluronic acid) within the previous 30 days, or theinability to complete the study.

### Study Design

**Figure 1:**
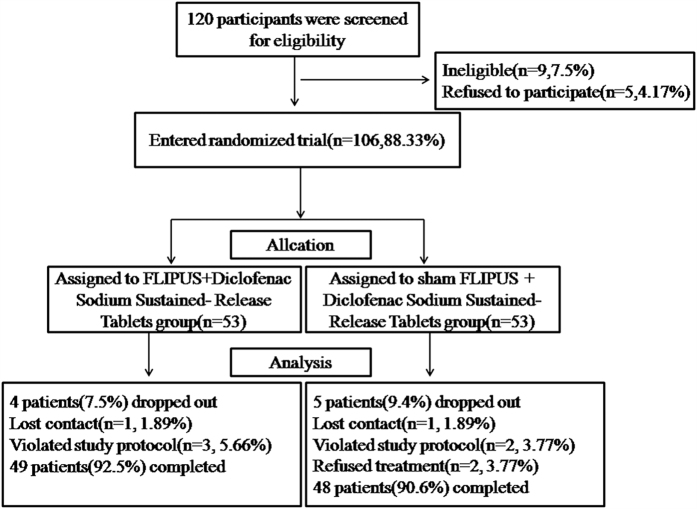
CONSORT diagram showing the disposition of patients in the study.

This pilot study was designed according to the CONSORT 2010 statement^27^. The study procedure is outlined in Fig. 1. All participants who fulfilled the study design criteria were assessed. Specifically, a detailed medical history was taken, a detailed physical examination was performed, and patients were questioned about their age, sex, weight, height, and duration of knee OA. Participants were assigned into group I (FLIPUS + diclofenac sodium sustained-release tablets) or group II (sham FLIPUS + diclofenac sodium sustained-release tablets) at a 1:1 ratio in a random and double-blinded manner. Randomization was performed using a computer generated list of random numbers, and was stratified by gender to ensure equal numbers of males and females in each group. A statistician who was unaware of the enrollment status assigned participants consecutively to treatment codes that corresponded to labels on otherwise identical concealed containers. Participants, investigators, and outcome assessors were blinded to the treatment for the duration of the study. Treatment assignments were not revealed prior to data collection and analysis.

**Figure 2:**
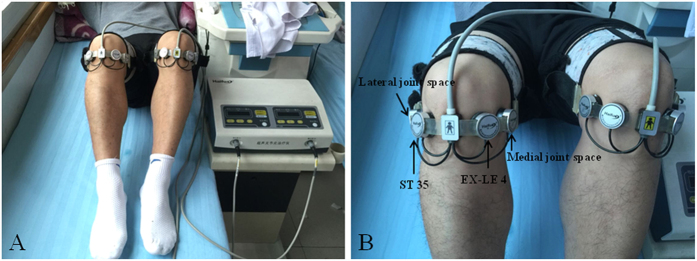
Procedure used for FLIPUS. The ST-35, EX-LE 4, interior, and lateral knee joint spaces have been marked and the heads have been fixed to ST 35, EX-LE 4, and the knee joint space.

No physiotherapy and pharmacotherapy were given prior to US treatment to either of the groups. US treatment was applied to both sides of the knee. Group I received FLIPUS for 20 min once daily fora total treatment duration of 10 days. Group II received a sham treatment (without energy output) for 20 min for the same treatment period. In low-intensity pulsed US mode, energy output or not, patients wouldn’t feel heat or any sensation. Patients and the US device were separated from each other by a curtain so that patients couldn’t see the power switch position. These measures were intended to enhanceblinding of the patients. All treatments were standardized using a device that placed the participant in a supine position, and the knee was angled ~90° at the flexion position. The four US probes were close to the surface skin of the ST 35 acupoint (located in the depression lateral to the patellar ligament when the knee is flexed), EX-LE 4 acupoint (located in the depression medial to the patellar ligament when the knee is flexed), and interior and lateral knee joint spaces, respectively (Fig. 2). The cartilage of lateral and medial femoral condyle was the tissue being targeted. The Model CZG200 Ultrasound Therapeutic Device for Arthritis used (Chongqing Haifu Medical Technology Co. Ltd., China) had an ultrasonic transducer diameter of 25 mm, a radius-of-curvature of 28 mm, a frequency of 0.6 MHz, a pulse repetition frequency of 300 Hz, a spatial and temporal average intensity (I_sta_) of 120 mW/cm^2^, and a duty cycle of 20%^28^,^29^. The ellipsoid-shaped acoustic focus was 0.25 mm in diameter and 0.54 mm in length, measured at the full width at half-maximum of the acoustic intensity^30^. At the same time, US therapy patients in Groups I and II received 75 mg oral sustained-release diclofenac sodium tablets (Beijing Novartis Pharma Ltd., China) once daily for the entire 10 day treatment period^31^.

### Outcome Measures

Clinical assessments of the participants were performed at baseline, after 10 days of treatment, and at follow-up after 4 and 12 weeks. The primary outcome was knee pain on movement for 5 minutes, as assessed using the visual analogue scale (VAS). The VAS instrument consisted of a 10-cm horizontal or vertical lines, a score of 0 cm indicated no pain, whereas 10 cm indicated very severe pain^32^. The secondary study outcomes were kneefunctional ability (assessed using the Chinese version of the Western Ontario and McMaster Universities Osteoarthritis Index WOMAC score), disability (assessed using Lequesne index LI), ambulation activity (ambulation speed AS), joint motion (active range of motion ROM), and HRQoL (assessed using the Medical Outcomes Study 36 Short-Form Health Survey SF-36).

The active ROM was measured with plastic goniometer with 25-cm movable double arms, marked in 1-degree increments. This device is reportedly reliable if the patient remains in one position for all measurements^33^. Measurement of knee flexion was performed in the supine position by simultaneously flexing the hip and knee, with the foot on the measured side resting on the table as far as possible. The fully extended knee was considered zero position, and the degrees of maximum flexion, maximum extension, and extension deficit, were recorded. A negative ROM score for extension indicated that the patient was unable to reach the zero position. The angle between maximum flexion and maximum extension was described as the excursion range^22^.

The ambulation time required to walk a distance 50 meters as fast as possible was measured with a stopwatch and recorded in minutes.

### Sample size

Using an open preliminary study on 20 usual consecutive patients with knee OA we calculated that 95% could be improved in the group I and an estimated 75% could improve in the group II. Thus, if the agreed alpha risk is 5% and the beta risk 20%, 50 patients per group or 53 allowing for 5% loss to follow-up was required for this analysis.

### Statistical Analyses

Statistical analyses were performed using SPSS 19.0 (SPSS Inc., Chicago, IL, USA). Data are expressed as means ± SDs (standard deviation). The calculations were based on detecting a mean difference of a 2 cm minimally clinically important difference (MCID) on a 10 cm VAS assuming a standard deviation of 2 cm, a 2-tailed test, an alpha level of 0.05 and a desired power of 80%^34^. The sociodemographic characteristics of the groups were evaluated using Chi-square tests. Prior to comparisons we tested whether the data were normally distributed and the variances were equal. If so, paired t-sample *t*-tests were used to compare the pre- and post-treatment changes in each group. Student’s *t*-tests were used to compare the two groups. Independent samples *t*-tests were used forcomparisons of the before and after treatment changes between groups, including the mean differences between groups, with 95% confidence intervals (CIs). If the data were not normally distributed non-parametric, Wilcoxon and Mann-Whitney U tests were applied. *P*-values < 0.05 were considered to be statistically significant.

## Results

### Patient Characteristics

One-hundred-and-twenty knee OA patients were screened for eligibility (see Fig. 1 for the CONSORT flow diagram). Nine of those (7.5%) screened declined to participate (failed to complete the evaluation and were not interested in treatment). Finally, 106 (recruitment rates: 88.3%) knee OA participants entered the study and underwent randomization. The patients were assigned randomly to group I (n = 53) or group II (n = 53). Ninety-seven (91.5%) of the 106 patients completed the double-blind phase, and nine (8.5%) dropped out (group I, n = 4; 7.5%; group II, n = 5; 9.4%). Their reasons for discontinuation were; loss to follow-up for an unknown reason (group I, n = 1; group II, n = 1), refused treatment (group I, n = 0; group II, n = 2), and violation of the study protocol (group I, n = 3; group II, n = 2). No FLIPUS-related adverse events were reported in either treatment group. There were no significant differences between the group I and group II with respect to age, gender, body mass index (BMI), duration of knee OA, blood pressure,glucose, and Kellgren & Lawrence class rating at baseline (*p* > 0.05; Table 1).

### Primary Outcomes

106 knee OA participants were included in the outcomes analyses and 97 patients completed follow-up. According to intention to treat analyses (ITT), minimally clinically important improvement (reduction in VAS scores more than 2 cm) was observed in 97 patients (91.5% in total, 92.5% in group I and 90.6% in group II respectively). There were no significant differences between groups with respect to VAS at baseline. The VAS measurements improved significantly in both groups after intervention (*p* = 0.000). However, the VAS reduction in group I was greater than in group II after 10 days of intervention (*p* = 0.000; Table 2).

### Secondary Outcomes

There were no significant differences between groups I and group II with respect to WOMAC score, LI, AS, ROM, and SF-36 subscale scoresat baseline. Most secondary measures improved from baseline after both interventions (*p* *<* 0.05). However, the reduction in WOMAC and LI scores was greater in group I than group II after 10 days of intervention (*p* = 0.001 and 0.000, respectively). There was a significantly greater improvement in the ambulation speed in group I compared with group II (*p* = 0.006). Although there was no difference between the two groups with respect to range of knee motion (*p* = 0.066) after 10 days of intervention, the total mean increment of range of motion was greater in group I than group II (*p* = 0.001). There was a significantly greater improvement in the SF-36 subscale scores in group I compared with group II (*p* *<* 0.05),except for the physical role and emotional role subscale scores (*p* = 0.066; Table 2).

### Change at Follow-up

The movement pain (VAS scores) and disability (LI scores) measures remained significantly lower at the 12-week follow-up compared with baseline in group I, but were higher than at baseline in group II (*p* = 0.000). The significantly greater improvements in VAS and LI scores were evident in group I compared with group II at both 4 and 12 weeks follow-up (*p* *<* 0.05; Table 3).

### Adverse Events

Minor and short lived NSAID (diclofenac sodium)-related adverse events were reported during the interventions. In group I these included headache and dizziness (2%), vertigo (2%), and gastrointestinal symptoms (13%). In group II these included vertigo (2%), transaminase elevation (2%), and gastrointestinal symptoms (15%). There were no significant differences between the two groups with respect to NSAID (diclofenac sodium)-related adverse, and no FLIPUS-related adverse events were found during and after interventions in either group.

## Discussion

OA is caused by bone breakdown and cartilage degeneration, including fibrosis, cracks, ulcers, and even loss of the full thickness of the articular cartilage^28^. Compared with drugs or surgery, US is preferred for its non-invasiveness, minimal adverse effects, and cost-effectiveness. Despite the popularity of US therapy for the treatment of knee OA, its clinical efficacy is somewhat controversial^35^.

The biological actions of US may vary widely depending on the physical parameters of US. In previous studies continuous ultrasonic waves with frequencies of 1 or 1.5 MHz^20^,^23^,^36^,^37^,^38^,^39^ and 1–2.5 W/cm^2 ^20,23,36,37,38,39 were applied. Knee OA not only affects the articular cartilage, but also involves the entire joint including the subchondral bone, synovial membrane, ligaments, joint capsule, and periarticular muscles. Therefore, previous studies selected the tendons and muscles around the knee joint for unfocused US application, including locations of tendinopathy and enthesopathy^23^,^37^,^39^. The biological effects of unfocused US are mainly considered to be thermal, which alleviates various muscles and tendons spasm^38^. However, the thermal effects of US for alleviating muscle and tendon spasms are limited for knee OA^38^. Muscle does not absorb energy well because of its homogeneity, high water content, and low collagen content, and heating muscles and tendons involves treating a larger area than unfocused US can heat effectively^38^. Although cartilage degeneration is the primary problem in knee OA, in clinical practice few studies have focused US therapy on articular cartilage directly. Although the pressurewaves propagated by US transfer mechanical energy into tissue^40^,^41^, a traditional unfocused US therapeutic regimen using higher frequency (≥1 MHz) ultrasonic waves barely propagates through the bone and fails to deliver US energy into the articular cartilage. In addition, energy from unfocused US can diffuse and destroy adjacentstructures^42^.

Interestingly, our previous *in vivo* experiment^29^ demonstrated that the main biological effects of FLIPUS (using a spatial and temporal mean intensity of 120 mW/cm^2^, a frequency of 0.6 MHz, a pulse repetition frequency of 300 Hz, and a 20% duty cycle) are mechanical, which could be quite different from the biological effects of US reported in other previous studies (thermal effects). We also revealed that FLIPUS at 0.6 MHz could propagate through the patella andsoft tissue to stimulate the cartilage directly, and also protect cartilage by decreasing the joint effusion volume, pro-inflammatory mediators, cell apoptosis, and also inducing cell proliferation^29^. Recently, pulsed US applied using a low intensity (<1 W/cm^2^) and low frequency (<1 MHz) had a positive effect on patients with knee OA, including alleviating joint symptoms, relieving joint swelling, increasing joint mobility, and reducing inflammation^28^. However, in clinical practice fewinvestigations have been conducted into the application of FLIPUS to the degenerated cartilage of patients with knee OA. Therefore, the current randomized, double-blind, placebo-controlled trial was conducted to evaluate the effectiveness of FLIPUS for the treatment of patients with symptomatic knee OA. This is the first study investigating the use of FLIPUS for degenerative cartilage in a clinical trial setting. Because of the controversial clinical efficacy of US, we used NSAIDs in both groups to avoid any potential nonintervention since the AAOS recommends NSAIDs for patients with symptomatic knee OA.

In addition to the physical parameters of FLIPUS, selecting the location for ultrasonic energy application is another key consideration with treatment. Previous studies have suggested that ST35 is a common point for treating pain in the knee joints and knee-related disorders^43^,^44^,^45^,^46^, whereas EX-LE4 is used for the treatment of both knee pain and knee arthritis^47^. The interior and lateral knee joint space affords an adequate acoustic channel that allows ultrasonic energy to be transferred to the surface of the intra-articular degenerative cartilage. In the current study both pain and joint function improved, after total treatment duration and follow-up after 3 months, in only patients that received FLIPUS + diclofenac sodium sustained-release tablets. Compared with sham, patients receiving FLIPUS treatment showed statistically significant improvements in all pain, knee functional ability, disability, ambulation activity, and HRQoL measurements. There were no FLIPUS-related adverse events during or after interventions. Based on these results, we conclude that FLIPUS therapy is a safe and effective treatment modality for pain relief and improving functions and HRQoL in patients with symptomatic knee OA.

Individuals with knee OA often complain of joint pain, stiffness, and difficulty with purposeful movement. Pain is not only the predominant symptom of knee OA, but also the main reason for medical consultation. The pain could be caused by several conditions, including loss of articular cartilage, capsular distension by effusion that leads to mechanical pain^48^, patellar and associated syndromes such as anserine bursitis or prepatellar bursitis due to inflammatory mediators and inflammatory pain^49^, and micro fractures and subchondral fractures^22^. In the current study the most striking effect of FLIPUS was pain reduction, which is consistent with previous studies^21^,^23^,^24^,^50^. In the current study the VAS pain scores improved in both groups. However, the VAS reduction was greater in group I than group II both after 10 days of intervention and during the follow-up period. In our results, small differences were found between groups (5.44 ± 0.84 vs 4.48 ± 0.84) because minor changes of VAS scores were detected when patients feel low pain after treatment^51^ (VAS ≤ 3/10, VAS scores were 1.54 ± 0.81 vs 2.28 ± 1.01 at endpoint respectively). The lower the severity of patient pain, the less obvious changes of VAS scores are found. We believe FLIPUS + NSAIDs could relieve pain more intensely than NSAIDs alone from the clinical significance we found, though small differences were found between groups at endpoint. We believe a number of ultrasonic effects can relieve pain. First, FLIPUS significantly increases extracellular matrix (ECM) production by downregulating chondrocyte apoptosis and upregulating cell proliferation, which both improve ECM preservation^29^. Second, FLIPUS can reduce effusion volumes to relieve mechanical pain^29^. Finally, FLIPUS might attenuate the release of inflammatory mediators (prostaglandin E2 and nitric oxide) and keeping them low over time, which could relieve inflammatory pain^29^.

Ambulation speed and joint ROM are important indicators of functional performance^39^, and restricted flexion of the knee appears to be an important determinant of disability in patients with OA^52^. The soft tissue around the knee OA may become fibrotic, contracted, or shortened when subjected to immobilization or inactivity due to joint pain, thereby decreasing the ROM and decreasing the patient’s ability to walk^22^. A previous study found strong correlations between knee joint ROM and disability^53^. Furthermore, restricted flexion of the knees was a strong risk factor for locomotor disability during activities including walking and climbing stairs^54^. Previous studies suggested the use of WOMAC for assessing the functional ability of the knee in patients with knee OA^55^,^56^, and the culturally and linguistically validated Chinese version of the WOMAC for mainland China was psychometrically robust in its validity, reliability, and sensitivity to change for patients with knee OA^57^.

The LI has been validated and is used for assessing kneedisability in patients with knee OA^58^. In the current study, the improvement in functional and disability status according to WOMAC and LI scores was significantly different in both groups after treatment compared with at baseline. However, the reduction in WOMAC and LI scores was greater in group I compared with group II both after 10 days of intervention and during follow-up. We also found that significantly greater improvements in ambulation speed and increment range of motion were evident in group I compared with group II. We hypothesize that the improved function and disability were attributable to the ability of ultrasonic waves to relieve pain. Therefore, the combination of FLIPUS and NSAIDs had better analgesia effects than NSAIDs alone.

Because of pain, loss of joint function, anddeformities, patients with knee OA have been reported to gradually reduce their physical activity, which consequently worsens their quality of life^59^,^60^. In the current study, HRQoL was assessed in knee OA patients using SF-36. The SF-36 was originally developed as an instrument for health surveying, and it has been used widely as a sensitive health status measure for clinical evaluation^61^. The SF-36 contains eight domains: the first four (physical function, physical role, bodily pain, and general health perceptions) assess physical health, and the last four (vitality, social function, emotional role, and mental health) assess mental health^62^. Several previous studies have revealed that pain severity, disability, and loss of joint function are negatively associated with quality of life in patients with OA^61^,^62^. In the current study there were significant differences between the two groups in six domains (general health, physical function, social function, bodily pain, vitality, and mental health) in SF-36. This suggests that FLIPUS and NSAIDs could improve HRQoL in patients with knee OA significantly compared with NSAIDs alone. Improvements in the HRQoL of patients with knee OA are due to pain relief and improved functional ability after the application of FLIPUS. In addition, the current data showed that there were no differences between the two groups with respect to the physical role and emotional role sub-scale scores because, even in the presence of severe joint pain and disability, the patients maintained their work activities and domestic chores during the study period.

No adverse effects have been reported with US in previous trials, and the current data suggest that US therapy is safe^20^,^35^,^50^. Similarly, no adverse events occurred during or after the FLIPUS treatment in the current study; therefore FLIPUS can be used safely in patients with knee OA.

In the current study, FLIPUS proved to be a safe and effective treatment modality for relieving pain, however,it has become clear that one cannot conclude a pain treatment is clinically significant based strictlyon statistical significance^63^. As such, the minimal clinically important difference (MCID) of VAS has been accounted for. The minimal detectable change (MDC) scores for the VAS in the group I was very high at the end of the treatment and 4 weeks later; the scores were greater than 4.5 cm. This change was greater than that of the minimal clinically important difference of 2.0 cm^64^ and our results are clinically significant. However, MDC scores for the VAS in both groups were lower than 2 cm 12 weeks later. These results indicated pain reduction of FLIPUS lasted for about 4 weeks after treatment.

The present study has some limitations that should be discussed. First, all participants included this study were enrolled from a single center, and there was a relatively small sample size. Therefore, future studies with larger populations and a multi-center clinical trial are needed. Second, the long-term effectiveness of FLIPUS should also be assessed in a continuing study. Third, we compared effectiveness between combination of FLIPUS + NSAIDs and NSAIDs in current study. The difference effectiveness between FLIPUS and NSAIDs on the functional status of patients with KOA is not clear which will be investigated in our future continuing study. Finally, the physical parameters of FLIPUS used in this trial were based on our previous *in vivo* experiment^37^, and an important direction for future research is elucidating the optimum acoustic exposure parameters of FLIPUS in patients.

In conclusion, the current study revealed that FLIPUS is a safe and effective treatment modality that causes pain relief and improves function and HRQoL in patients with knee OA. This study makes a contribution that may have important implications for future US research, particularly in terms of managing knee pain, stiffness, physical function, and HRQoL in knee OA.

## Additional Information

**How to cite this article**: Jia, L. *et al*. Efficacy of focused low-intensity pulsed ultrasound therapy for the management of knee osteoarthritis: a randomized, double blind, placebo-controlled trial. *Sci. Rep.* **6**, 35453; doi: 10.1038/srep35453 (2016).

## Figures and Tables

**Table 1:** Baseline Demographic and Clinical Characteristics.

Variable	FLIPUS + NSAIDs	Sham FLIPUS + NSAIDs	*P*-value
n	53	53	
Sex (M/F)	14/39	16/37	0.666
Age, y	63.42 ± 9.73	61.34 ± 10.25	0.774
BMI, kg/m^2^	25.79 ± 3.46	26.17 ± 5.92	0.568
Duration of knee OA, month	140.36 ± 87.32	137.45 ± 92.48	0.532
SBP, mmHg	125.65 ± 8.25	126.35 ± 9.79	0.676
DBP, mmHg	75.45 ± 7.85	76.38 ± 7.78	0.725
Glucose, mmol/L	9.59 ± 8.24	9.69 ± 7.87	0.458
Kellgren & Lawrenceclass rating			
Grade II	46(86.79%)	44(83.02%)	0.587
Grade III	7(13.21%)	9(16.98%)	

NOTE: Data expressed as mean ± SD

Abbreviations: BMI = body mass index; SBP = systolic blood pressure; DBP = diastolic blood pressure.

**Table 2:** Primary and Secondary Outcomes Mean Differences in Change From Baseline.

Variable	Baseline		Endpoint		Between-Group Difference	
	Group I	Group II	Group I	Group II	Mean* 95%CI	*p*
n	53	53	49	48		
**Primary Outcome**						
VAS	6.98 ± 1.06	6.76 ± 1.02	1.54 ± 0.81	2.28 ± 1.01		0.000
VAS mean change from baseline 95%CI			5.44 ± 0.84 5.05 to 5.79	4.48 ± 0.84 4.08 to 4.88	0.96 0.63 to1.29	0.000
**Secondary Outcome**						
WOMAC	44.34 ± 10.79	42.42 ± 9.39	10.92 ± 8.57	15.88 ± 5.26		0.001
WOMAC mean change from baseline 95%CI			33.42 ± 7.99 29.55 to 37.29	26.54 ± 5.85 23.52 to 29.56	6.88 4.10 to 9.66	0.000
LI	7.56 ± 2.73	7.10 ± 2.12	1.82 ± 1.44	2.66 ± 1.12		0.000
LI mean change from baseline 95%CI			5.74 ± 1.99 4.87 to 6.61	4.44 ± 1.39 3.57 to 5.02	1.30 0.62 to 1.98	0.000
ROM,degree	127.42 ± 6.36	127.68 ± 6.75	130.78 ± 5.20	129.14 ± 6.27		0.066
ROM mean change from baseline 95%CI			−3.36 ± 2.95 −5.56 to−1.05	−1.46 ± 1.97 −4.05 to 1.13	1.90 0.90 to 2.90	0.001
AS, m/min	0.61 ± 0.05	0.65 ± 0.29	0.91 ± 0.26	0.74 ± 0.32		0.006
AS mean change from baseline 95%CI			−0.30 ± 0.16 −0.39 to −0.21	−0.12 ± 0.12 −0.32 to 0.01	0.18 0.13 to 0.24	0.000
SF-36: GH	40.64 ± 13.58	43.14 ± 17.12	57.22 ± 10.37	45.60 ± 16.55		0.007
GH mean change from baseline 95%CI			16.58 ± 9.29 −18.39 to −2.21)	2.46 ± 5.68 3.09 to −1.51	14.12 8.38to19.89	0.000
SF-36: PF	54.30 ± 12.12	57.60 ± 14.75	81.20 ± 11.50	73.00 ± 11.56		0.000
PF mean change from baseline 95%CI			−26.90 ± 13.32 −29.89 to −6.31	−15.40 ± 12.32 −18.37to −4.68	11.50 6.41 to16.59	0.000
SF-36: RP	38.50 ± 29.11	42.90 ± 29.90	51.50 ± 26.37	55.23 ± 19.72		0.873
RP mean change from baseline 95%CI			−13.00 ± 16.24 −8.36 to −19.36	−12.33 ± 17.69 −7.76to −18.82	0.67 0.15 to13.64	0.631
SF-36: RE	48.67 ± 22.14	43.33 ± 13.88	63.20 ± 33.16	58.21 ± 25.52		0.485
RE mean change from baseline 95%CI			−14.53 ± 12.13 −19.36 to −10.36	−14.88 ± 25.03 −20.16 to −12.64	−0.35 −1.07 to 8.10	0.803
SF-36: SF	54.75 ± 14.70	51.75 ± 13.83	81.00 ± 15.00	71.25 ± 14.34		0.001
SF mean change from baseline 95%CI			−26.25 ± 17.34 −32.14 to −20.36	−19.50 ± 15.19 −25.09 to−13.91	6.75 0.28 to 13.22	0.047
SF-36: BP	31.30 ± 13.03	34.46 ± 13.11	68.31 ± 11.56	55.22 ± 11.32		0.000
BP mean change from baseline 95%CI			−37.01 ± 14.44 −41.90 to−32.12	−20.76 ± 9.49 −31.43 to−12.52	16.25 11.39 to21.11	0.000
SF-36:Vitality	44.00 ± 15.12	40.80 ± 11.44	65.62 ± 11.39	56.70 ± 10.86		0.000
Vitality mean change from baseline 95%CI			−21.62 ± 12.35 −26.93− to −16.31	−15.90 ± 9.41 −20.33 to− 11.47	5.72 1.36 to 10.07	0.024
SF-36: MH	42.64 ± 13.51	41.36 ± 10.74	68.06 ± 10.45	59.60 ± 10.39		0.000
MH mean change from baseline 95%CI			−23.64 ± 12.42 −31.65 to−20.11	−18.66 ± 7.55 **-**22.48 to−14.84	4.98 0.90 to 9.06	0.007

^*^Mean difference between groups in change from baseline scores.

NOTE. Values are mean ± SD Abbreviations: VAS = visual analog scale; WOMAC = Western Ontario and McMaster Universities Osteoarthritis Index; LI = Lequesne index; ROM = range of motion; AS = ambulation speed; SF-36 = short form 36 item general health questionnaire; GH = general health; PF = physical function; RP = role physical; RE = role emotional; SF = social function; BP = bodily pain; MH = mental health.

**Table 3:** VAS and LI score measures after 4 and 12 weeks of follow-up.

	VAS scores				LI sores			
	Baseline	4 weeks	12 weeks	*P*-value	Baseline	4 weeks	12 weeks	*P*-value
FLIPUS + NSAIDs	6.98 ± 1.06	2.36 ± 1.22	6.42 ± 1.57	0.000	7.56 ± 2.73	2.76 ± 1.71	6.78 ± 2.48	0.000
Sham FLIPUS + NSAIDs	6.76 ± 1.02	4.12 ± 0.75	7.18 ± 0.94	0.000	7.10 ± 2.12	3.96 ± 0.90	7.84 ± 1.56	0.000
*P*-value	0.396	0.000	0.007		0.460	0.000	0.006	

NOTE. Values are mean ± SD. Abbreviations: VAS = visual analog scale; LI = Lequesne index.
